# Monitoring of Low Levels of Furfural in Power Transformer Oil with a Sensor System Based on a POF-MIP Platform

**DOI:** 10.3390/s150408499

**Published:** 2015-04-13

**Authors:** Nunzio Cennamo, Letizia De Maria, Girolamo D’Agostino, Luigi Zeni, Maria Pesavento

**Affiliations:** 1 Department of Industrial and Information Engineering, Second University of Naples, Via Roma 29, Aversa 81031, Italy; E-Mail: luigi.zeni@unina2.it; 2 Department of Transmission and Distribution Technologies, RSE Research on Energetic System S.p.A, Via Rubattino 54, Milano 20134, Italy; E-Mail: letizia.demaria@rse-web.it; 3 Department of Chemistry, University of Pavia, Via Taramelli, Pavia 12-27100, Italy; E-Mails: girolamo.dagostino@unipv.it (G.D.); maria.pesavento@unipv.it (M.P.)

**Keywords:** furfural (furan-2-carbaldehyde), power transformer, insulating oil system, molecularly imprinted polymer, plastic optical fiber, surface plasmon resonance, optical sensor

## Abstract

In this work an innovative, miniaturized and low cost optical chemical sensor (POF-MIP platform), based on a molecular imprinted polymer (MIP) and surface plasmon resonance in a plastic optical fiber (POF), is presented and preliminarily tested for monitoring of furfural (furan-2-carbaldehyde) in transformer oil. To this end, the optical platform was coupled to an MIP layer, highly selective for furfural. The ability of the developed sensor to directly detect furfural in the insulating oil was investigated. The detection limit of the sensor has been found to be 9 ppb, with a linear response up to about 30 ppb. However there is a sensible response up to 0.15 ppm. Because of the small linearity range, the Hill equation is suggested for the quantification. The sensor has been effectively tested in real oil samples collected from aged electrical equipment removed from service. The assessed concentration of furfural is in good agreement with that evaluated by a high pressure liquid chromatography (HLPC) method, confirming the good selectivity of the proposed sensor.

## 1. Introduction

The power transformer is a key component of the Electric Transmission and Distribution (T&D) system. Its integrity assessment is very complex but essential to avoid irreversible damages with consequent heavy impacts on maintenance costs and on T&D network services, due to outages. Among causes which can lead to a transformer failure (*i.e.*, hot spots, partial discharges), the accelerated degradation of its solid insulating system, *i.e.*, oil impregnated cellulosic insulation materials, strongly depends on the operating condition of the transformer [1,2]. Actually, sometimes the degradation of its dielectric parts begins much earlier than the designed end-of-life of the transformer (foreseen as 30 years), due to an accelerated thermal aging of both the insulating oil and the paper, but while the regeneration of a degraded insulating oil is possible by appropriate treatments or even by its replacement with a new compatible oil [3], the refurbishment of degraded paper requires invasive and costly operations that must be necessarily performed by the manufacturer, because it can involve the total replacement of transformer windings. For this reason it is well established [4,5] that the end of useful life of a transformer is mainly determined by thermal deterioration of papers and that a careful monitoring of parameters linked to this process is of fundamental importance for utilities to check the “health” of the  in-service transformers.

One of the main components of the insulation paper (*i.e.*, Kraft paper) is cellulose, which consists of a long linear chain of β-D glucose molecules [5,6]. As the paper ages, its mechanical strength changes significantly because of degradation (depolymerization) of the cellulose chains; in a domino effect, the decrease of mechanical strength also reduces the ability of the transformer to withstand short circuit stress, confirming that these two properties are not mutually disjoint but are in synergy. For this reason, the mechanical strength of the paper is considered an important diagnostic parameter in transformers and its reduction down to 50% is assumed as an indication of the end-of-life of a transformer [4].

The mechanical performance of the cellulose is defined by the degree of polymerization (DP) of the paper, which corresponds to the average number of glucosidic (β-D glucose) units in a single chain of cellulose [4,6]. During the aging of paper, the DP parameter decreases from values of 1000 ÷ 1200 (new paper) to 150 ÷ 100 (degraded and fragile paper). The viscometric test, according to the IEC 60450 [7], provides a reliable and direct measure of the DP value but actually this method is not practical, because it requires a sample of paper from the transformer insulation system, which is impossible to access during regular operation. The indirect measure of the insulating paper decomposition products dissolved in oil, such as carbon dioxide and carbon monoxide (CO_2_ and CO) [8,9], methanol [4,10], furfural (furan-2-carbaldehyde, 2-FAL) and related furans is generally preferred. While CO and CO_2_ can be generated by decomposition of the oil during long term oxidation too, methanol, furfural and furans are only formed by the processes of thermal degradation of the cellulose [4,8,11]. Their concentration in transformer oil is strictly correlated to the degree of polymerization of the paper [4,8,9], as for instance in the case of Kraft insulating paper. Although methanol has been widely demonstrated to be very promising as a marker of ageing [4,10,11], currently the 2-FAL concentration is used worldwide as one of the main indicators to estimate the ageing of the paper in a transformer [4,8]. As the presence of furan compounds in oil is not related to the degradation of the oil itself, they can be used as chemical markers in transformer insulating oil to assess the overall DP with a high degree of confidence [8,12].

For typical power transformers containing Kraft insulating paper, it is generally assumed that a furan content in the range between 0.1 and 1.0 ppm infers a DP decrease between 700 and 450, equivalent to a moderate deterioration of these electrical equipment and thus an early warning of degradation of paper insulation.

Currently, common practice consists of periodic oil sampling from in-service transformers and in analysis usually performed in a laboratory remote from the sampling site. It is carried out usually by chromatographic methods, such as for instance by high pressure liquid chromatography (HPLC) according to the IEC 61198 method [13]. This technique is quite complex, requiring the extraction of the substances of interest from the sample before the injection on the chromatographic column, the use of expensive equipment and specialized operators both for acquisition and for data analysis, and a long time period for obtaining the results. Normally, these measurements are scheduled on an annual basis and only in case of critical situations is an intensification of the oil sampling required. Recently methods or devices alternative to HLPC have been reported in the literature. These are mainly based on electrochemical techniques, but they are not very sensitive and not highly selective [14,15]. In particular, those based on amperometric techniques are potentially subject to noise and to electromagnetic interferences and therefore they are not suitable for an on-line application. Moreover, the electrochemical measurements require the presence of electrolytes to improve the electrical conduction of the system, so that the original sample must be modified before measurement, most often by complex extraction procedures [15]. To overcome these problems a new approach, exploiting surface plasmon resonance (SPR) on plastic optical fiber and molecularly imprinted polymers (MIP), is proposed in this work.

Recently, SPR biosensors in optical fibers have been shown to be able to play an important role in numerous important fields, including pharmaceutical research, medical diagnostics, environmental monitoring, food safety and security, where fast, portable, low cost and rugged units are needed for early detection and identification [16,17]. In general, the optical fiber employed is either a glass one or a plastic one (POF). For low-cost SPR sensing systems, POFs are especially advantageous due to their excellent flexibility, easy manipulation, great numerical aperture, large diameter, and the fact that plastic is able to withstand smaller bend radii than glass. The advantage of using POFs is that the main features of POFs, that have increased their popularity and competitiveness for telecommunications, are exactly those that are important for optical sensors based on glass optical fibers, with the addition of simpler manufacturing and handling procedures [18,19].

The required selectivity can be obtained in different ways by using a receptor layer very specific for the considered analyte in contact with the SPR active surface. Most often it consists of a thin layer or even a monolayer of biological receptor molecules such as for example antibodies. Molecularly imprinted polymers (MIPs) are synthetic receptors obtained by molecular imprinting methods [20,21], presenting a number of favorable aspects for sensing in comparison to bioreceptors, including a better stability out of the native environment, reproducibility and low cost. A further reason for using chemical instead of biological receptors, in the particular application examined herein, is that the measurements should be done directly in the sample, *i.e.*, in a mineral oil phase, in which the most usual biological receptors do not perform at their best. On the contrary, MIPs should present higher affinity and selectivity for the substrate in non-aqueous media [22,23]. This combined sensing approach, based on a POF-MIP sensor, could be extremely suitable for on-line application. Nevertheless, only a few sensors have been found in the previous literature combining MIPs with an optical fiber [19,24,25]. As a proof of principle furfural (2-FAL) has been considered among several furanic compounds possibly present in used transformer oils, since it is usually the most prominent component of paper decomposition in power transformer. In principle, similar sensors for different furanic compounds of interest in the transformer ageing control could be prepared in the same way. The feasibility of the sensor, and its applicability in oil samples, has been preliminarily tested in synthetic solutions [26]. In the present investigation it was applied to “real life” samples, *i.e.*, exhausted transformer oils.

## 2. Experimental Section and Material and Methods

### 2.1. POF-MIP Platform and Experimental Setup

The optical platform was home-made in three steps, as previously described extensively [18,19]. First the cladding of a plastic optical fiber (D-type plastic optical fiber) was removed along half its circumference, then on the exposed core a layer of Microposit S1813 photoresist was spin coated, and finally a thin gold film was sputtered using a sputtering machine. The plastic optical fiber has a PMMA core of 980 µm and a fluorinated polymer cladding of 20 µm. The final thickness of the photoresist buffer was about 1.5 µm and that of the gold film, which presented a good adhesion to the photoresist buffer layer, was 60 nm.

**Figure 1:**
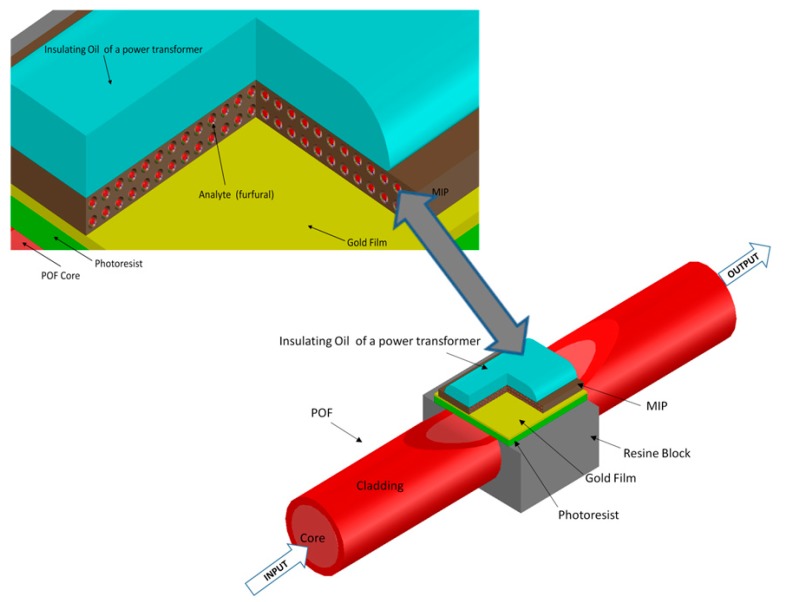
Optical Sensor system with MIP layer. Inset: Cross section of the MIP sensing region.

The realized region was about 10 mm long, over which an MIP layer, very specific for 2-FAL, can be easily realized as reported below. Figure 1 schematically shows the optical sensor system (POF-MIP platform). The experimental setup was arranged to measure the transmitted light spectrum and included a halogen lamp, illuminating the POF chemical sensor, and a spectrum analyzer. The employed halogen lamp (Model no. HL-2000-LL, manufactured by Ocean Optics, Dunedin, FL, USA) exhibits a wavelength emission range from 360 nm to 1700 nm, while the spectrum analyzer detection range is from about 330 nm to 1100 nm (an Ocean Optics USB2000+VIS-NIR-ES spectrometer has been employed). The spectrometer is finally connected to a computer. The SPR curves along with data values are displayed on-line on the computer screen and saved with the help of advanced software provided by Ocean Optics.

### 2.2. MIP Sensing Layer

The gold planar surface over POF (SPR active surface) was washed with ethanol and then dried in a thermostatic oven at 60 °C prior to deposition of the sensing layer, *i.e.*, a specific molecularly imprinted polymer (MIP) layer. A schematic view of the active surface cross section is shown in Figure 1.

The prepolymeric mixture for MIP was prepared according to the classical procedure reported in [19] with only slight modifications. Divinylbenzene (DVB), the cross-linker, was also the solvent in which the functional monomer (that is, methacrylic acid, MAA), and the template, furfural (2-FAL) are dissolved. The reagents were at molar ratio 1 (2-FAL):4 (MAA):40 (DVB). For example, a typical prepolymer mixture for the MIP specific for 2-FAL is composed of 20 µL of furfural, 80 µL of MAA and 1.4 mL of DVB. Notice that DVB is at the same time the cross linker and the solvent. The mixture was uniformly dispersed by sonication (visually homogeneous solution) and de-aerated with nitrogen for 10 min. Then the radical initiator AIBN (16 mg in the example described) was added to the mixture. Fifty µL of the prepolymeric mixture were dropped over the sensing region of the optical fiber and spun for 2 min at 1000 rpm. Thermal polymerization was then carried out for 16 h at 80 °C. The template was extracted by repeated washings with 96% ethanol.

### 2.3. Laboratory Procedure

The laboratory procedure used for the investigation of the sensor signal and for the standardization in oil samples was as follows: a small amount of oil, about 50 µL, was dropped over the MIP layer (sensing zone), as seen in Figure 1, and the spectrum recorded after only a few minutes incubation. SPR transmission spectra were normalized to the reference spectrum, *i.e.*, that obtained in air before spin coating the MIP layer, where no plasmon resonance takes place because of the refractive index of air.

For calibration, standard samples with known 2-FAL concentrations in fresh transformer oil were measured. The calibration curves were obtained by plotting the variation of the resonance wavelength (Δλ_ex_) as a function of the 2-FAL concentration. It was evaluated considering the transmission minimum in oil not containing 2-FAL as reference value.

The same single sensor was repeatedly used for preparing the calibration curve by briefly fluxing the active surface with ethanol between successive measurements. This cleaning procedure between successive measurements must be performed in order to avoid contamination, considering that the determination is carried out in a drop manually placed over the sensing region. When the measurements are performed via a flow cell based system, no cleaning with ethanol is required.

### 2.4. Oil Samples

In-service oil samples were considered to verify the response of the POF-MIP sensor in used oil. As a typical insulating oil, a Nytro Libra insulating oil from Nynas S.r.L. (Stockholm, Sweden) was considered. The used oil was collected from serviced electrical equipment, *i.e.*, two 145 kV oil insulated current transformer CTs (CT.134 and CT.729 respectively), which operated in different substations of the Italian Transmission System Operator. The transformers had been operating for about 30 years. The samples were taken from gas-tight syringes used for dissolved gas analysis (DGA) and for furan analysis, and were analyzed by an external laboratory. Furan concentrations were measured by means of high pressure liquid chromatography according to the IEC61189 method. Results confirmed the presence of degradation compounds generated over the years of transformer operation (Table 1). These products originate both from the oxidation of the oil and from the degradation of cellulose. The same oil, but a new sample, was used for sensor calibration.

**Table 1:** Composition of two used transformer oil samples from different substations of the Italian Transmission System Operator.

Chemical Agent	Method	Unit	CT.134	CT.729
Total gas concentration	IEC 60567	μL/L	78,579	65,611
Furfural (2-FAL)	IEC 61189	ppm	0.112	0.114
Σ furan derivatives		ppm	<0.010	<0.010
Methanol		ppm	0.567	0.408
Ethanol		ppm	0.306	0.250

As reported in Table 1, the two samples are very similar, and both CT.134 and CT.729 show a remarkable furfural (2-FAL) concentration (0.112 ppm and 0.114 ppm respectively). No other furan derivatives were detected. From Table 1 significant methanol and ethanol concentrations emerge as well. These compounds, like furans, are oil soluble by-products of the aging of oil-impregnated cellulose [4,5,6] and their presence could potentially interfere with the furan detection by the sensor.

## 3. Results and Discussion

### 3.1. Characterization of the POF-MIP Sensor for Furfural (2-FAL) Detection in Fresh Transformer Oil

**Figure 2:**
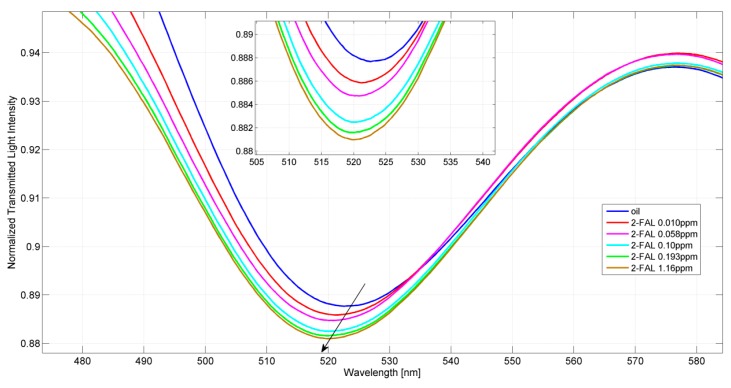
Normalized SPR spectra acquired by the POF-MIP sensor P1 for different furfural concentrations (in ppm) in fresh oil. Inset: zoom of resonance wavelengths.

Two POF-MIP sensors, namely sensor 1 (P1) and sensor 2 (P2), have been tested for measurements in a drop, as described in the Experimental section. The transmission spectra, obtained with the POF-MIP sensor in the case of a widely used mineral transformer oil (Nytro Libra insulating oil), exhibit several transmission minima in the considered wavelength range (330–1100 nm). Nevertheless, the minimum at around 520 nm for P1 and 530 nm for P2 is much better defined than the other minima, and, moreover, it depends on the 2-FAL concentration. Figure 2 shows the SPR resonances obtained with the particular POF-MIP sensor P1. For better clarity the inset of Figure 2 reports only the resonance wavelengths, obtained for the standard samples at different concentrations of 2-FAL in fresh transformer oil [26]. It is clearly seen that the resonance wavelength is shifted to lower values (blue shift) when the 2-FAL concentration increases, indicating that 2-FAL effectively combines with MIP from the oil matrix here considered.

**Figure 3:**
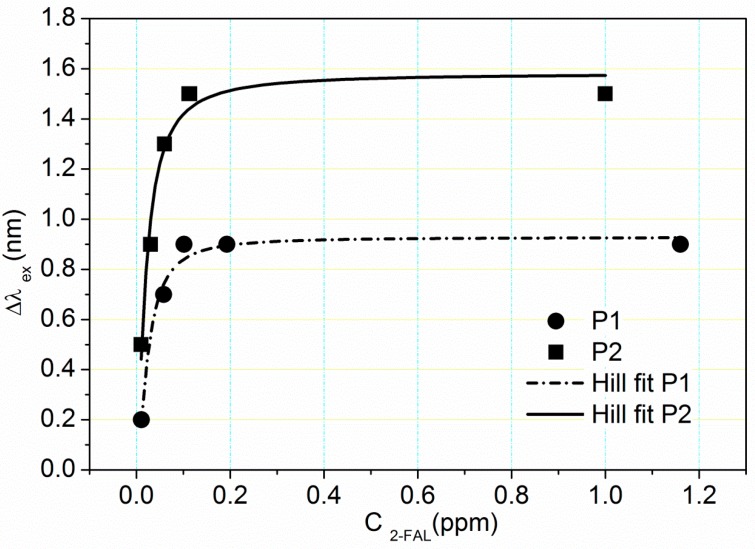
Absolute value of resonance wavelength shift versus the concentration of 2-FAL [ppm], obtained with two different POF-MIP sensors P1 and P2, and the fitting of the data by Hill equation.

Typical curves reporting the transmission minimum wavelength variation (Δλ_ex_) *versus* the concentration of 2-FAL, obtained with two different sensors are shown in Figure 3.

In both cases the function is not linear since the slope decreases at increasing concentration, reaching a zero value at sufficiently high 2-FAL concentration, about 0.15 ppm. Since Δλ_ex_ is directly proportional to the concentration [19,24] the deviation from the linearity must be ascribed to the saturation of the combination sites present in the molecularly imprinted polymer.

The affinity constant for the sites can be evaluated from the Langmuir model for the adsorption on a limited number of sites on a solid phase which takes place according to the following equilibrium:

(1) $$A+\bar{P}⇆\bar{AP}$$

where A indicates the analyte, P the interaction site in MIP obtained by the molecular imprinting procedure and AP the adduct. The barred species are those in the solid phase. The corresponding equilibrium constant (affinity constant) is:

(2) $$K_{f}=\frac{\left[\bar{AP}\right]}{\left[A\right]\left[\bar{P}\right]}$$

Considering that the total concentration of sites in the polymer phase is:

(3) $$c_{site}=\left[\bar{P}\right]+\lceil \bar{AP}\rceil$$

and that the instrumental response is directly proportional to the variation of the concentration of A in the polymer, the following relationship holds:

(4) $$∆\lambda_{ex}=k\lceil \bar{AP}\rceil$$

Thus the analytical response depends on the analyte concentration (c) according to:

(5) $$\text{Δ}\lambda_{ex}=\frac{kc_{site\;}{\left(K_{f}c\right)}^{h}}{1+{\left(K_{f}c\right)}^{h}}$$

The parameter *h* takes into account the possible lack of homogeneity of the interaction of the substrate with the solid. In the original Langmuir isotherm it is equal to 1, *i.e.*, not any dishomogeneity is considered. The final relationship (5) is similar to the Hill equation which is widely used for the response of biosensors [27]. The parameters of the Langmuir Equation (5), obtained by the commercially available Software OriginPro 8.5 by Origin Lab Corp. (Northampton, MA, USA) are reported for the two sensor considered in Table 2. The program returns the reciprocal of *K*_f_, *i.e.*, the dissociation constant *K*_d_. The fitting to the experimental values is presented in Figure 3.

**Table 2:** Parameters of the Hill equation for two different POF-MIP sensors for 2-FAL. Standard samples were prepared in fresh transformer oil.

POF-MIP Sensor	Δλ_lim_ (nm)	*K*_d_** (ppm)	*h*	Adj R-Square
P1	0.92 (5)	0.024 (5)	1.6 (3)	0.9677
P2	1.58 (0.09)	0.020 (3)	1.3 (3)	0.9506

Notice that in both cases *h* is not significantly different from 1, considering the high error associated. The value of *K*_d_, the dissociation constant, is about 0.02 ppm in both the considered examples, which is sufficiently low to make it possible the detection at low concentration level. *K*_d_ is similar for the two considered sensors, as expected because the same imprinted polymer is used. The corresponding affinity constant *K*_f_ is 5 × 10^6^ mol^−1^ L. It is more than two orders of magnitude higher than those previously determined for other small molecules, such as TNT and nicotine, with the corresponding MIP of the same composition [19,24] which have been found to be near 10^4^ mol^−1^ L. This is certainly due in part to the different molecular structure of the site, but probably most importantly to the fact that in the system here considered the sample medium is oil, instead of water as in the previously investigated cases. Notice that according to Equation (5) the concentration for which Δλ_ex_ = Δλ_lim_/2 corresponds to the dissociation constant *K*_d_, *i.e.*, to the reciprocal of the affinity constant *K*_f_.

The maximum value of the signal (Δλ_lim_) is significantly different in the two sensors. This is obtained when (*K*_f_*c*)*^h^* is much higher than 1, *i.e.*, c is much higher than *K*_d_, or much lower than *K*_f_. In that situation:

(6) $$\text{Δ}\lambda_{ex}=\text{Δ}\lambda_{lim}=kc_{site}$$

The two sensors here examined have a similar linearity range of the standardization curve, only up to about 30 ppb. However Δλ_lim_ is higher for P2 than for P1 indicating a different concentration of sites in the polymer, and/or a different sensitivity of the detection method, as seen from Equations (5) and (6). Evidently the plasmon resonance is different in each individual sensor, due for example to differences in the thickness of the gold layer or of the fiber cladding. Such irreproducibility could be particularly relevant since each sensor is built up by hand, so a certain degree of irreproducibility must be expected.

The standardization curve should be a straight line for c much lower than *K*_f_. The regression lines at these conditions are here reported:

$$∆\lambda =27\left(5\right)\cdot c_{2-FAL}\;+0.03\;\left(3\right)\;\;\;\;R^{2\;}=0.9449\;\;\text{for P1}$$

$$∆\lambda =28\;\left(7\right)\cdot c_{2-FAL}+0.08\;\left(13\right)\;\;\;\;R^{2\;}=0.9367\;\;\;\text{for P2}$$

The lower detection limits determined from the “almost linear” part of the standardization curve at lower concentrations is about 0.002 ppm for P1 and 0.009 ppm for P2. The lower detection limits are evaluated from twice the standard deviation of the solution at zero concentration (blank sample). This value is close to the lower detection limits required by the official control methods for transformer oil [13], so the detection method based on POF-MIP sensor can be interesting for on line preliminary controls.

### 3.2. Furfural in Ex-Service Transformer Oil Samples

The detection of furfural was assessed in real oil samples collected from two ex-service current transformers (CT.134 and CT.729). The insulating oil of these aged electrical devices is the same used for the previous assessment of the SPR sensor response. In Table 1 the concentration of some degradation products generated over years of transformers operation is reported. These products originate both from the oil oxidation, as for example gases, and from the degradation of cellulose, like alcohols and furfural (2-FAL).

**Figure 4:**
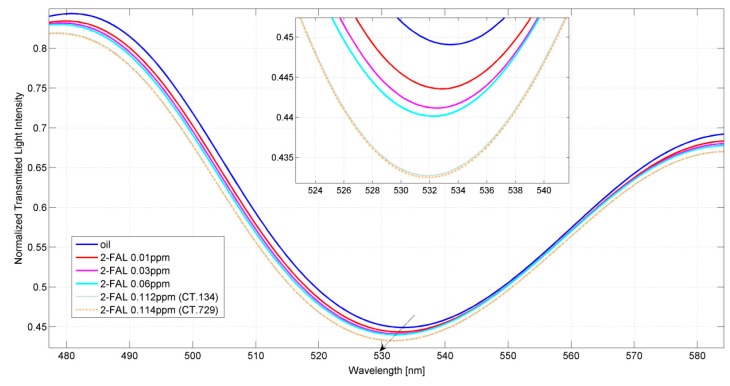
Normalized SPR spectra acquired by the POF-MIP sensor P2 for different furfural (2-FAL) concentrations in ppm in fresh oil and in two serviced transformer oils (CT.134 and CT.729). Inset: zoom of resonance wavelengths.

Figure 4 compares the transmission spectra at around 530 nm, obtained with fresh oil standards, and the spectra obtained with the CT.134 and the CT.729 oil samples directly contacted with the sensing surface (sensor P2) without any treatment or dilution. The transmission minimum wavelength is somewhat different from that reported in Figure 2, which was relative to a different sensor (P1). It is clearly seen from Figure 4 that the resonance wavelength is blue shifted at increasing concentration of 2-FAL in fresh oil. The spectra obtained with the two used oil samples are very similar to each other as expected because the 2-FAL concentration is similar (see Table 1) and they are blue-shifted with respect to that obtained in fresh oil, because of the presence of 2-FAL. The similarity of the transmission spectra in new and used oil samples shows that no interference from other compounds, deriving from the decomposition of paper or oil, occurs when using the POF-MIP sensor here proposed.

For both the samples considered the analytical response was Δλ_ex_ = 1.5 nm.  Considering the non-linearity of the sensor response, the concentrations of the samples can be evaluated by the Hill equation [27], once the parameters are known, according to the following relationship deriving from Equation (5):

(7) $$c_{2-\text{FAL}}=\frac{K_{d}}{\sqrt[{h}]{\frac{∆\lambda_{lim}}{∆\lambda_{ex}}-1}}$$

Using the parameters of the Hill equation reported in Table 2, the concentration was evaluated to be 0.17 ppm, with an uncertainty of 0.06 ppm, so the concentration found is not significantly different from that determined by the chromatographic analysis. The uncertainty is high, probably because the concentration is very near to the high detection limit. As the concentration determined by the sensor is in agreement with that determined independently, it must be deduced that the many other substances present in the samples, for example those reported in Table 1, do not interfere with the sensor response. This shows the very good selectivity of the proposed device in the considered samples, probably due to the high selectivity of the considered artificial receptor (MIP). The uncertainty is noticeably high, but it is sufficient for screening purposes, and in any case it could be improved simply by diluting the sample in order to bring the concentration well within the detection range.

## 4. Conclusions

In this work an optical device based on an SPR excitation in a POF platform has been described for the measurement of very low levels (˂0.15 ppm) of furfural (2-FAL) in transformer oils. The sensing layer in the developed POF-MIP sensor is a molecularly imprinted polymer specific for 2-FAL. It has been demonstrated to work properly in the oil medium in which 2-FAL must be detected, even better than in water solution, for example, as far as the affinity constant is concerned. Selective and rapid responses, for which small amounts of sample are required, are obtained. Moreover the POF-MIP sensor is robust, cheap and suitable for frequent measurements, possibly by housing the sensor body in a flow cell, through which the insulating oil could be forced to perform on-line measurements on transformers. Evidence of the feasibility of this approach emerged from tests carried out on samples of ex-service insulating oil. No interference from the usual components of used transformer oils, either coming from the oil or cellulose degradation, could be detected.

Notice that the proposed measurement procedure is very convenient and only requires a small amount of oil. Even if it was here applied to used oil from ex-service transformers, it could be used to the determination of 2-FAL in oil inside the transformer, due to the very small oil volume required for the determination. This could be drawn out of the transformer by a flowing circuit similar to that commonly used for the determination of gaseous degradation products. Alternatively the measurement could be done by placing the sensor directly inside the flowing circuit.
